# Magnetic Transition to Antiferromagnetic Phase in Gadolinium Substituted Topological Insulator Bi_2_Te_3_

**DOI:** 10.1038/srep10309

**Published:** 2015-05-14

**Authors:** Jinsu Kim, Kyujoon Lee, Toshiro Takabatake, Hanchul Kim, Miyoung Kim, Myung-Hwa Jung

**Affiliations:** 1 Department of Physics, Sogang University, Seoul 121-742, Republic of Korea; 2 Department of Quantum Matter, ADSM, Hiroshima University, Higashi-Hiroshima 739-8530, Japan; 3 Department of Nano Physics, Sookmyung Women’s University, Seoul 140-742, Republic of Korea

## Abstract

There are many interests to achieve long-range magnetic order in topological insulators of Bi_2_Se_3_ or Bi_2_Te_3_ by doping magnetic transition metals such as Fe and Mn. The transition metals act as not only magnetic dopants but also electric dopants because they are usually divalent. However, if the doping elements are rare-earth metals such as Gd, which are trivalent, only magnetic moments can be introduced. We fabricated single crystals of Bi_2-x_Gd_x_Te_3_ (0 ≤ × ≤ 0.2), in which we observed magnetic phase change from paramagnetic (PM) to antiferromagnetic (AFM) phase by increasing x. This PM-to-AFM phase transition agrees with the density functional theory calculations showing a weak and short-ranged Gd-Gd AFM coupling via the intervening Te ions. The critical point corresponding to the magnetic phase transition is x = 0.09, where large linear magnetoresistance and highly anisotropic Shubnikov-de Haas oscillations are observed. These results are discussed with two-dimensional properties of topological surface state electrons.

The spin-momentum locked surface states of three-dimensional (3D) topological insulators (TIs) are protected by time reversal symmetry (TRS)^1^,^2^,^3^,^4^,^5^. The gapless surface states give rise to exotic phenomena such as the quantum spin Hall effect^1^ and a large linear magnetoresistance (MR) induced by two-dimensional (2D) Dirac electrons^6^. However, if magnetic ordering is introduced into TIs, the TRS can be broken. This leads to anomalous quantum phenomena such as a quantum anomalous Hall (QAH) effect^7^,^8^, image magnetic monopoles^9^, and giant magneto optical effects^10^. Introducing magnetic elements into TIs is advantageous to investigate the correlation between topological state and magnetism. Several papers reported that the magnetic impurities such as Fe or Mn can break the TRS and affect the gapless surface states, leading to a gap opening^11^,^12^,^13^,^14^. However, such magnetic impurities induce electrical doping effect at the same time, i.e., the charge carriers are doped. When the magnetic impurity elements are transition metals such as Fe and Mn, which are mostly divalent, the substitution of Fe^2+^ or Mn^2+^ for Bi^3+^ creates hole dopants into the system. Therefore, it is hard to detect the effect only from the magnetic ordering on non-trivial topological properties. On the other hand, when the magnetic elements are rare-earth metals such as Ce and Gd, which are trivalent, no charge carriers are doped but only magnetic moments can be introduced. Among the rare-earth elements, Gd has a large magnetic moment with half-filled 4f electrons which are seven unpaired electrons. So the substitution of Bi by Gd may maximize the magnetic doping effect on TIs. One theoretical paper proposed that the single quintuple layer of GdBiTe_3_ film can be a QAH insulator with ferromagnetic ordering, and the gapless chiral edge states exist inside the bulk band gap^15^. However, experimentally GdBiTe_3_ has not been achieved in a single phase form 16. In addition, there are some controversial reports on the solubility limit of Gd in Bi_2_Te_3_ (i.e. Bi_2-x_Gd_x_Te_3_)^17^,^18^,^19^,^20^. Bulk samples revealed that the equilibrium solubility is less than x = 0.10^18^, while thin-film samples revealed that the maximum Gd concentration is x = 0.80^19^,^20^.

In this study, we fabricate the single crystals of Bi_2-x_Gd_x_Te_3_ (x = 0, 0.06, 0.08, 0.09, 0.12, 0.15, and 0.20) and report the effects of Gd substitution on the topological properties in Bi_2-x_Gd_x_Te_3_. From the magnetic data, we obtain the phase diagram of Bi_2-x_Gd_x_Te_3_ as a function of x. The Gd substitution drives the magnetic phase transition from paramagnetic (PM) phase to antiferromagnetic (AFM) phase around x = 0.09, where the magnetic interaction is strongly suppressed. This result indicates the presence of a magnetic critical point (MCP) near x = 0.09 and simultaneously suggests an abrupt change of electronic and magnetic properties around x = 0.09. The density functional theory calculations well explain this PM-to-AFM phase transition, which is due to weak Gd-Gd AFM coupling via the intervening Te ions. The electronic transport experiments reveal various intriguing properties such as high electrical resistivity, large linear magnetoresistance, and highly anisotropic Shubnikov-de Haas (SdH) oscillations in the x = 0.09 sample.

## Results and Discussion

**Figure 1:**
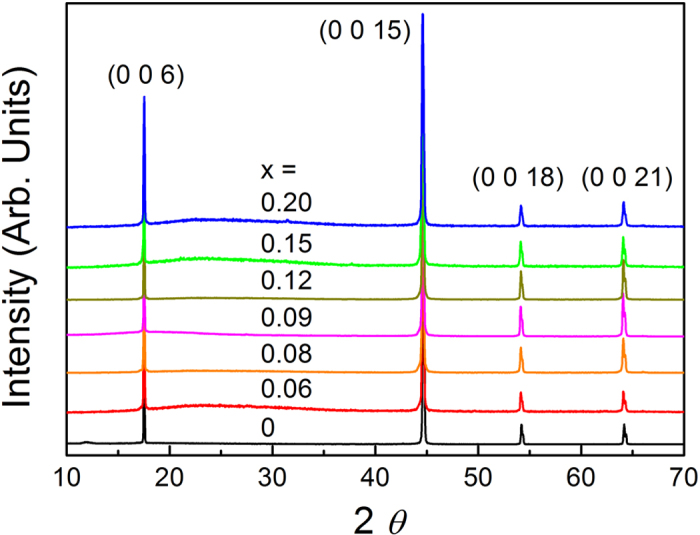
Single-crystal X-ray diffraction patterns for Bi_2-x_Gd_x_Te_3_.

The samples of Bi_2-x_Gd_x_Te_3_ were characterized by X-ray diffraction (XRD) patterns. Figure 1 shows the single-crystal XRD data, where the peak positions of (003) family reflections are observed for all samples. This reveals that Bi_2-x_Gd_x_Te_3_ crystallizes in the same structure as Bi_2_Te_3_. The lattice parameters are obtained to be *a* = 4.39 ± 0.01 Å and *c* = 30.48 ± 0.04 Å. We also measured powder XRD patterns, which are not shown here, to check possible existence of impurities or secondary phases. The most possible secondary phase is Gd_2_Te_3_ mixed with Bi_2_Te_3_^19^. However, no secondary peaks are observed in our single-crystal and powder XRD data. In addition, the XRD peak positions do not shift with x, indicating that the lattice parameters are not changed by the Gd substitution. This result is consistent with the previous reports^20^, where no change of lattice parameters is found even at x = 0.6. This may be caused by small difference of crystal ionic sizes between Gd and Bi.

**Figure 2:**
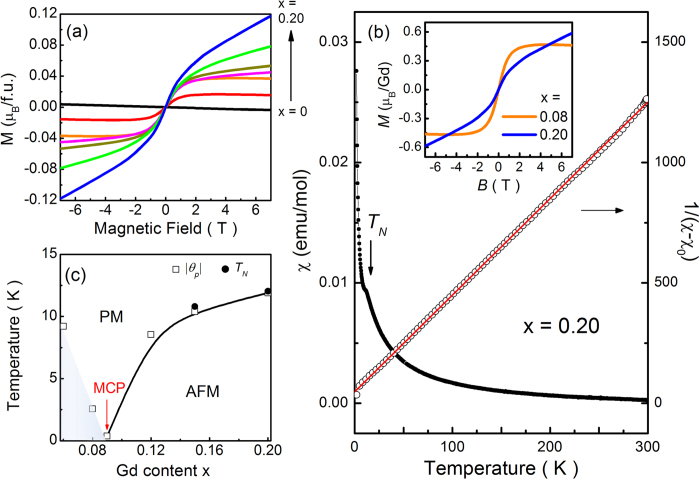
Magnetic properties of Bi_2-x_Gd_x_Te3. (**a**) Magnetization versus field M(H) measured at 2 K. (**b**) Magnetic susceptibility versus temperature χ(T) measured at 1 kOe for x = 0.20. Its inverse curve is displayed in the right axis. The arrow represents the antiferromagnetic transition temperature of T_N_ and the red line represents the fitted curve with the Curie-Weiss law. In the inset, the M(H) data for x = 0.08 and 0.20 are replotted with a scale of μ_B_ per Gd. (**c**) Phase diagram with the magnetic transition from paramagnetic (PM) and antiferromagnetic (AFM) phases. In the PM phase, there is an additional regime with ferromagnetic (FM) exchange interaction. The magnetic critical point (MCP) regime is displayed around x = 0.09. The open squares represent the Weiss temperature θ_P_ obtained from the Curie-Weiss law and the closed circles represent the antiferromagnetic transition temperature T_N_ obtained from the χ(T) data.

In order to check if the Gd elements are effectively well substituted, we have measured the magnetization M(H) at 2 K by applying the magnetic field parallel to the *c* plane. The results are displayed in Fig. 2(a) for Bi_2-x_Gd_x_Te_3_. The magnetic moment increases with increasing x, demonstrating that the Gd elements are effectively well substituted into Bi_2_Te_3_. Although similar M(H) signals are observed, we can divide the magnetization curves into two groups; one of x ≤ 0.08 and the other of x ≥ 0.09. The curves for x ≤ 0.08 show the existence of the diamagnetism originated from the parent compound Bi_2_Te_3_. In addition to the diamagnetism, there is an abruptly increasing feature of magnetic moment at low fields. This indicates an existence of weak FM and/or PM component for x ≤ 0.08. Since we cannot observe any magnetic hysteresis, the magnetic ground state is PM. On the other hand, for x ≥ 0.09 the abruptly increasing feature of magnetic moment is suppressed, and the diverging signals of magnetic moment are still alive up to 7 T. The magnetic moment is not saturated in fields up to 7 T, indicating another magnetic ground state of x ≥ 0.09. For easy comparison, the representative curves of x = 0.08 and 0.20 are replotted in the inset of Fig. 2(b). Here it should be mentioned that the absolute value of M(H) is one order of magnitude smaller than that expected for free Gd^3+^ ion, unlike those reported in other Gd-substituted Bi_2_Te_3_ systems^17^,^19^,^20^. This may be attributed to strong magnetic anisotropy. Thereby the M(H) curves with the magnetic field along the c axis are also measured. The magnetic moments are slightly enhanced but not more than two times. We are confident that the low magnetic moment is an intrinsic property of Gd-substituted Bi_2_Te_3_. Further experiments such as neutron scattering and μSR measurements are needed to validate the origin of the low Gd moment.

To address the origin of the magnetic exchange interaction, we have also measured the magnetic susceptibility χ(T) as a function of temperature for all the samples. The representative curve of χ(T) for x = 0.20 is shown in Fig. 2(b). There is an anomalous peak structure around T_N_ = 12.0 K, which reveals that the AFM phase transition occurs at T_N_. Also in the x = 0.15 sample, the similar structure is observed with different temperature of T_N_ = 10.8 K. Such an AFM exchange interaction was reported in some films of Bi_1.8_Gd_0.2_Te_3_ and Bi_1.4_Gd_0.6_Te_3_^19^,^20^, where slightly negative Weiss temperatures of θ_P_ = −1.3 K and −2.5 K represent the existence of weakly AFM ordering although the long-range AFM transition was not observed at temperatures down to 5 K. Except this AFM feature, all the χ(T) data are well fitted by the Curie-Weiss law, χ(T) = χ_0_+C/(T-θ_p_), where χ_0_ represents the temperature-independent contribution including the van-Vleck paramagnetism and the core diamagnetism, C is the Curie constant, and θ_p_ is the Weiss temperature. The linear plots of 1/(χ-χ_0_) in the high temperature range give two important fitting parameters of C and θ_P_, which are related with the effective magnetic moment and the magnetic exchange interaction, respectively. From this analysis, we can obtain the reasonable effective magnetic moment per Gd of μ_eff_  = 7.54, 8.39, 8.18, 7.33, 7.34, and 7.06 μ_B_ for x = 0.06, 0.08, 0.09, 0.12, 0.15, and 0.20, respectively. These μ_eff_ values are close to the theoretical value of 8.0 μ_B_ expected for free Gd^3+^ ion. These results indicate that the Gd ion is effectively well substituted with the stoichiometric composition rate of x. More importantly, we can obtain the Weiss temperature of θ_P_ = 9.21, 2.56, -0.38, −8.55, -10.4, and −11.9 K for x = 0.06, 0.08, 0.09, 0.12, 0.15, and 0.20, respectively. The positive θ_P_ value decreases with increasing x (<0.09) and then changes the sign to negative on further increase of x (≥ 0.09). These results suggest that the magnetic exchange interaction is changed from FM to AFM coupling at the boundary of x = 0.09, where the Weiss temperature is close to zero. Furthermore, the results are consistent with the M(H) data. As aforementioned, the M(H) data showed an abrupt increase at low fields for the samples of x < 0.09, which is suppressed for the samples of x > 0.09. The critical composition of the magnetic transition seems to be x = 0.09, and thereby we plot the phase diagram in Fig. 2(c) based on the χ(T) analysis. Even though we cannot judge the onset temperature of FM exchange interaction, it is clear to be magnetic phase change from PM with FM coupling at x < 0.09 to AFM ordering at x > 0.09. Here it should be pointed out that the Weiss temperatures (θ_P_ = −10.4, and −11.9 K) for x = 0.15 and 0.20 are well matched with the Neel temperatures of T_N_ = 10.8 and 12.0 K, respectively. In Fig. 2(c), the composition of x = 0.09 is marked as MCP judging from the magnetic phase transition.

In order to understand the magnetic properties of Bi_2-x_Gd_x_Te_3_, we have performed the density functional theory (DFT) calculations^21^,^22^ in a spin-polarized manner using the *Vienna ab initio simulation package* (VASP)^23^. The exchange-correlation interaction was treated within the generalized gradient approximation^24^ and the ions were represented by the projector-augmented-wave potential^25^,^26^. The Gd 4f orbitals were described by including the on-site Coulomb repulsion, U =7.0 eV, following the Dudarev’s scheme^27^. The plane wave basis set was used within the kinetic energy cutoff (E_cutoff_) of 200 eV, and the convergence with respect to the basis set size was examined by increasing E_cutoff_ of up to 400 eV. The Brillouin zone (BZ) integration was performed by using the **k**-point mesh equivalent to the 8 ×8 ×2 grid in the BZ of the hexagonal unit cell. We have employed a 4 ×4 ×1 hexagonal supercell, i.e., Bi_96_Te_144_ containing 48 formula units of the Bi_2_Te_3_, to examine the interaction between the substitutional Gd_Bi_’s. For all the investigated configurations, we used the experimental lattice constants (a = 4.3830 Å and c = 30.487 Å)^28^ and the atomic structures were relaxed to ensure that the Hellmann-Feynman forces are smaller than 0.02 eV/Å.

First of all, we found a weak and short-ranged AFM coupling between the magnetic moments of two Gd_Bi_’s. The total energy difference between the FM and the AFM configurations (ΔE = E^FM^ – E^AFM^) was 8.9 meV for the two Gd_Bi_’s occupying the first nearest neighbor (NN) sites in a quintuple layer (QL), and was 2.5 meV for the two Gd_Bi_’s occupying the second and the third NN sites in a QL. The ΔE’s were negligible (or order-of-magnitude smaller) when the two Gd_Bi_’s are further apart in a QL or located in different QLs. This is consistent with the previous theoretical report of a negligible interaction between substitutional Mn’s residing in different QLs^29^. The first NN Gd_Bi_ pair is located on the *same* Bi layer in a QL sharing two Te atoms (one on the central Te layer and the other on the outer Te layer). In the second (third) NN Gd_Bi_ pair configuration, two (one) Te atoms make bonds with one Gd_Bi_ on a Bi layer and the second Gd_Bi_ on the other Bi layer of the same QL. Thus, the AFM coupling seems to be achieved due to a superexchange interaction between Gd^3+^ ions via intervening Te atom(s) as suggested in ref. 18.

At low concentration, the Gd_Bi_’s will tend to disperse uniformly in the host material Bi_2_Te_3_ due to a kinetic barrier for diffusion, resulting in a negligible concentration of the 1^st^ - 3^rd^ NN Gd_Bi_ pair configurations. Then, most of the magnetic moments of Gd atoms will show random distribution without any magnetic ordering, which corresponds to a PM state. As the concentration increases, the probability for two Gd_Bi_’s to occupy the first, the second, and the third NN sites would increase. Consequently, the sample with higher Gd concentration is more likely to develop the AFM ordering. In order to support this expectation, we performed the total energy calculations for the configurations containing four and six Gd atoms in the 4 ×4×1 hexagonal supercell. Among the different (nine) four-Gd-atom configurations corresponding to x =0.083, the linear alignment of Gd_Bi_’s in a single Bi layer of a QL is calculated to be the most stable. For the linear chain of Gd_Bi_’s, there are two almost degenerate magnetic structures with the total energy difference smaller than 0.2 meV: the AFM (…↑↑↓↓…) ordering and the ferrimagnetic (…↑↑↑↓…) ordering. These magnetic orderings are more stable than the FM ordering, and ΔE is 4.7 and 4.9 meV for the AFM and the ferromagnetic ordering, respectively. These findings imply that the concentration x =0.083 is close to the phase transition point (or MCP) from PM to AFM. As for the six-Gd-atom case (corresponding to x =0.125), on the other hand, a hexagonal arrangement of Gd_Bi_’s in a single Bi layer of a QL is the most stable configuration and it shows a perfect AFM ordering. This AFM phase is lower in energy than the FM phase by 14.7 meV. These results are in accordance with the experimental observation of the PM phase for x < 0.09 and the AFM phase for x = 0.15 as shown in Fig. 2(c).

**Figure 3:**
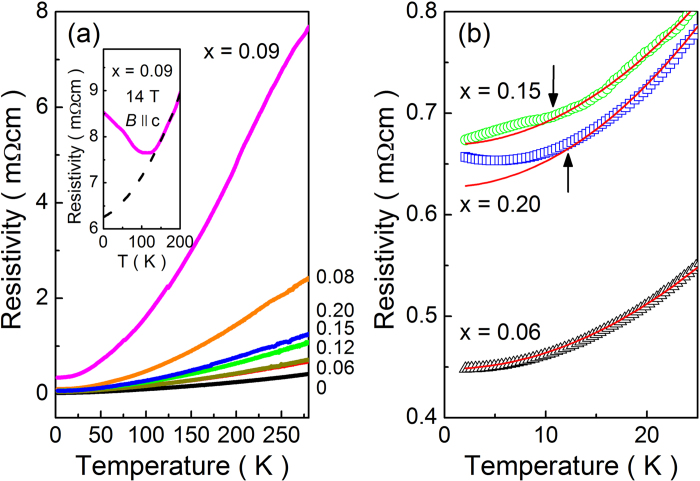
Electrical resistivity versus temperature ρ(T) for Bi_2-x_Gd_x_Te_3_. (**a**) The inset shows the ρ(T) data measured in an applied field of 14 T for x = 0.09, and the dashed line represents the Fermi-liquid behavior proportional to the square of temperature. (**b**) Low temperature data of ρ(T) for Bi_2-x_Gd_x_Te_3_. The arrows indicate the Neel temperature T_N_ = 10.8 and 12.0 K for x = 0.15 and x = 0.20, respectively. The solid red lines represent the T^2^ dependence of Fermi liquid behavior.

The magnetic correlation is developed from the PM to AFM phase as increasing x, and the MCP of x = 0.09 is determined by the magnetic phase transition. More information on the MCP can be obtained from the transport experiments. Figure 3 shows the electrical resistivity ρ(T) as a function of temperature measured with a conventional four-probe method. A simple metallic behavior is observed for all the samples. The absolute value of ρ(T) increases with increasing x, except x = 0.09 that is the MCP. As seen in the inset of Fig. 3(a) for the x = 0.09 sample, the in-field resistivity shows a sudden upturn below 120 K and thereby deviates from the Fermi-liquid behavior proportional to the square of temperature (T^2^). Similar behavior was reported in Te excessive Bi_2_Te_3_ crystal, where this upturn was attributed to the magnetothermoelectric effect^30^. The applied magnetic field gives rise to the change of carrier type from n- to p-type at low temperatures. This hints that the Fermi level shifts to lower energy by applying magnetic field. If we conjecture that the Fermi level is located at the boundary of conduction band edge, which will be treated later, the magnetic field could cause the Fermi energy to be in the bulk band gap. It should be noted that this sudden upturn is detected only for x = 0.09, that is the MCP. In addition, when the low-temperature resistivity data are magnified, one can see a clear sign of magnetic ordering in the x = 0.15 and 0.20 samples. As seen in Fig. 3(b), there are clear deviations from the T^2^ dependence of the Fermi-liquid behavior below T_N_ = 10.8 and 12.0 K for x = 0.15 and 0.20, respectively. For comparison, in the x = 0.06 sample where no magnetic ordering is found, the resistivity is simply proportional to T^2^.

**Figure 4:**
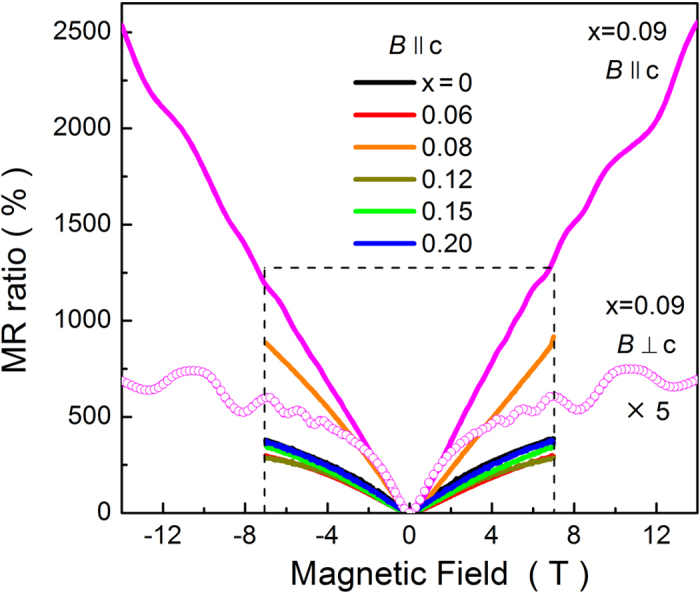
Magnetoresistance ratio data measured at 2 K for Bi_2-x_Gd_x_Te_3_. Solid lines represent the MR data taken with applying magnetic field along the c axis (H // c). For comparison, we plot the MR data of x = 0.09, which were measured in the H ⊥ c configuration and multiplied by 5.

Such a sudden increment of ρ(T) gives indication of giant magnetoresistance at low temperatures. Thus, we have measured the magnetoresistance ρ(H) at 2 K and plot the magnetoresistance ratio MR = [ρ(H)-ρ(0)]/ρ(0) in Fig. 4. In general, all the MR curves are positive, meaning that the in-field resistivity is larger than the zero-field resistivity. The MR curves behave like normal metallic systems, i.e., they are quadratic at low fields and tend to saturate at high fields^6^,^31^,^32^. The MR values at 7 T are between 300 and 400%, except x = 0.09 in which the MR is enormously enhanced and linearly increased up to 2500% at 14 T. Such large and linear MR behavior was reported by many groups 6,33,34,35 and it was explained by the linear energy dispersion of 2D surface state of the TIs. This result infers that the Fermi level for x = 0.09 is located in the surface state regime. Also, it should be mentioned that the temperature dependence of in-field resistivity is non-metallic, although the zero-field resistivity shows a simple metallic behavior. The non-metallic transport behavior is one typical feature of topological insulating properties.

Now let us focus on the high-field MR data of x = 0.09. For comparison, in Fig. 4 we plot the MR data for x = 0.09 in both H // c and H ⊥ c configurations. The highly anisotropic MR signals possessing prominent SdH oscillations are observed. For H // c, the MR is enormously linear, as aforementioned. The period of SdH oscillation is F = 30.51 T, in good agreement with the previous result arising from the 2D surface state of Bi_2_Te_3_ (F = 33.3T)^33^. On the other hand, the MR signal for H ⊥ c is small and tends to saturate in the high field regime, which is a typical signature of the bulk conduction in TIs^6^,^33^. In addition, the SdH oscillation in the H ⊥ c configuration is different from that in the H // c configuration. The period of F = 21.5 T for H ⊥ c is close to the value obtained from the 3D bulk Fermi surface of Bi_2_Te_3_ (F = 23.3 T)^33^. These results are quite reasonable because the charge carriers in the 2D surface state can make circular motion in the plane normal to the c axis but cannot in the plane possessing the c axis. Therefore, we conclude that the enormously linear MR for H // c is related with the 2D surface state and the MR for H ⊥ c is associated with the 3D bulk band. Then, now we discuss all the intriguing results for x = 0.09, that is the MCP. Since the ρ(T) data show the simple metallic behavior with *n*-type charge carriers, but the magnetic field drives non-metallic behavior at low temperatures, the Fermi level for x = 0.09 should be located at the boundary of bulk conduction band edge. At the same time, it should be located in 2D surface state regime because the MR data show unusual features arising from the surface state carriers of TIs such as large linear MR and the SdH oscillations with the 2D origin. Moreover, the highly anisotropic MR signals with the SdH oscillations indicate that the Fermi surface should possess two characters from the 2D surface state and bulk conduction band. As a result, the Fermi level for the MCP can be conjectured to be in the bulk conduction band edge coexisting with the 2D surface state energy dispersions. These results are quite different from those reported in TIs substituted by divalent transition metals as magnetic impurities^11^,^12^,^13^,^14^, where the TRS is broken and thereby the non-trivial topological properties are not expected. However, in the substituted TIs with trivalent rare-earth metal of Gd, the topological properties are re-entrant at a certain amount of Gd. This may be related with the localized character of rear-earth metals with 4f magnetic moments.

## Conclusions

In conclusion, we grew *n*-type Bi_2-x_Gd_x_Te_3_ single crystals (x = 0, 0.06, 0.08, 0.09, 0.12, 0.15, and 0.20), and verified the crystallinity and homogeneity by using X-ray diffraction experiments. The actual Gd substitution rate was confirmed by the magnetization measurements. The magnetic phase transition from a PM phase to an AFM phase occurs at x = 0.09, where exotic features are observed. The x = 0.09 sample, the MCP, has a relatively large resistivity, and it shows a non-metallic behavior driven by magnetic field. Besides, large linear MR with highly anisotropic SdH oscillations, which originates from 2D surface state transport, is observed only for x = 0.09. From the analysis of such distinguishing properties at the MCP, the Fermi level lies around the bulk conduction band edge and crosses the surface band as well. These findings imply that the topological surface state can possibly coexist with localized magnetic impurity bands.

## Methods

The single crystals of Bi_2-x_Gd_x_Te_3_ were grown by simple melting method in a vertical tube furnace with local temperature gradient. Each element of the stoichiometric ratio was put into a cleaned quartz tube with a small amount of excessive Te and sealed in vacuum. We introduced 5% excessive Te to compensate the Te loss caused by its high vapor pressure. We obtained *n*-type charge carriers for all Bi_2-x_Gd_x_Te_3_ samples because the Te excess generated Te_Bi_-type antisite defects which unintentionally generate the *n*-type charge carriers in the system^36^,^37^,^38^. The crystals were grown and treated according to the temperature sequences; warming up to 800 °C, cooling down to 550 °C, and annealing at 550 °C for 3 days. All the obtained crystals were well cleaved with a shiny surface perpendicular to the c axis. The magnetic and transport properties were measured with the superconducting quantum interference device-vibrating sample magnetometer (SQUID-VSM) up to 7 T and the physical property measurement system (PPMS) up to 14 T.

## Author Contributions

J.K. and K.L. carried out all the measurements. J.K. and M.-H. J. wrote the main manuscript text and M.K. and H.K. wrote the theoretical part. The high-field data were taken by T.T. All authors reviewed the manuscript.

## Additional Information

**How to cite this article**: Kim, J. *et al*. Magnetic Transition to Antiferromagnetic Phase in Gadolinium Substituted Topological Insulator Bi_2_Te_3_. *Sci. Rep.* **5**, 10309; doi: 10.1038/srep10309 (2015).
